# Effects of Religious Fasting on Markers of Oxidative Status in Vitamin D-Deficient and Overweight Orthodox Nuns versus Implementation of Time-Restricted Eating in Lay Women from Central and Northern Greece

**DOI:** 10.3390/nu16193300

**Published:** 2024-09-29

**Authors:** Spyridon N. Karras, Konstantinos Michalakis, Fotios Tekos, Zoi Skaperda, Periklis Vardakas, Panayiotis D. Ziakas, Maria Kypraiou, Marios Anemoulis, Antonios Vlastos, Georgios Tzimagiorgis, Costas Haitoglou, Neoklis Georgopoulos, Evangelos G. Papanikolaou, Demetrios Kouretas

**Affiliations:** 1 Laboratory of Biological Chemistry, Medical School, Aristotle University, 55535 Thessaloniki, Greece; tzimagio@auth.gr (G.T.); haitoglu@auth.gr (C.H.); 2 Endocrine Practice, Department of Obesity and Metabolism, 11521 Athens, Greece; 3 Department of Biochemistry-Biotechnology, School of Health Sciences, University of Thessaly, 41500 Larissa, Greece; fotis.tek@gmail.com (F.T.); zoiskap94@gmail.com (Z.S.);; 4 Department of Medicine, University of Brown, Providence, RI 02903, USA; pd.ziakas@gmail.com; 5 Assisting Nature Centre of Reproduction and Genetics, 57001 Thessaloniki, Greece; 6 Medical School, Aristotle University, 55535 Thessaloniki, Greeceantonisvlastos1958@gmail.com (A.V.); 7 Division of Endocrinology, Department of Internal Medicine, School of Health Sciences, University of Patras, 26504 Patras, Greece; neoklisgeorgo@gmail.com

**Keywords:** oxidative stress, Mediterranean diet, time-restricted eating, Orthodox fasting

## Abstract

**Background/Objectives**: The Mediterranean diet has been widely suggested to exert significant beneficial effects on endothelial oxidative status and cardiometabolic health. Greek Orthodox monasteries, due to their specific nutritional and sartorial habits, comprise a population thatstrictly adheres to nutritional patterns with restricted eating and a plant-based subset of the Mediterranean diet, often accompanied by profound hypovitaminosis D. Time-restricted eating (TRE) is also adopted bya large part of the general lay Greek population for health-promoting reasons, without restrictions on animal product consumption, as imposed by Orthodox religious fasting. However, the comparative effects of these nutritional patterns on oxidative stress markers remain scarce. **Methods**: The present study attempted to evaluate the effects of Christian Orthodox fasting (COF) in a group of vitamin D-deficient and overweight Orthodox nuns from Central and Northern Greece compared to the implementation of TRE, a 16:8 dietary regimen (16 h of food abstinence and 8 h of feeding) in a cohort of adult women from the general population from the same region with regard to markers of endothelial oxidative status. A group of 50 women from two Orthodox monasteries in Northern Greece and one group of 50 healthy lay women were included. During the enrollment, a detailed recording of their dietary habits was performed, along with a scientific registry of their demographic and anthropometric characteristics (via bioimpedance). The Orthodox nuns followed a typical Orthodox fasting regimen [daily feeding window (8 a.m.–4 p.m.)], whereas the lay women followed a TRE 16:8 regimen with the same feeding time-window with a recommendation to follow a low-fat diet, without characteristics of the Mediterranean diet. We included a complete biochemical analysis, as well as calciotropic profiles [calcium—Ca, albumin, parathyroid hormone—PTH, and 25-hydroxyvitamin D—25(OH)D] and markers of TAC (total antioxidant capacity), GSH (glutathione),and thiobarbituric acid reactive substances (TBARSs) concentrations as markers of oxidative status. **Results**: All the groups were compared at the baseline regarding their calcium, PTH, and 25(OH)D concentrations, with no statistically significant differences between the groups apart from higher PTH levels in the nuns due to lower 25(OH)D levels. The Orthodox nuns manifested a lower median GSH compared to the controls (6.0 vs. 7.2, *p* 0.04) and a higher median TAC (0.92 vs. 0.77, *p* < 0.001). The TBARS comparisons showed no significant difference between the two groups. No significant associations of oxidative status with 25(OH)D, PTH, and the markers of glucose homeostasis were evident. **Conclusions**: The results of this small pilot study indicate that both dietary regimens have advantages over the oxidative markers compared to each other, with increased TAC in the group of Orthodox nuns after a 16-week period of COF compared to a 16:8 TRE and increased GSH concentrations in the lay women group. Future randomized trials are required to investigate the superiority or non-inferiority between these dietary patterns in the daily clinical setting.

## 1. Introduction

Christian Orthodox fasting (COF) is a vital subset of the Mediterranean diet (MD) [1,2,3,4], which for religious reasons is considered to be deeply integrated into the cultural dietary behavior of a large part of the Greek population [5,6,7,8,9] for prolonged periods (from 120 to 180 d) annually [8]. Orthodox monasteries follow this archetypal pattern of diet throughout the year, with periods of more strict fasting rituals 2–6 weeks before religious celebrations, as a meansof physical and mental prosperity and personal spiritual development [1,2]. However, besides the spiritual significance of COF, a plethora of cohort studies suggest that COF shares the beneficial effects of the typical MD by promoting specific cardioprotective mechanisms, including reduced intake of dietary cholesterol and fatty acids, thus providing optimal effects on plasma lipid concentrations [9,10]. These benefits have been mainly attributed to the integration of a plant-based diet along with characteristics of dietary restriction regardinganimal products (meat, dairy products, and eggs) [6] and the restriction of caloric intake during the COF periods [11,12,13,14]. We have previously reported on the beneficial effects of COF on the adipokine profile [7,8,14] as well as on glucose homeostasis in both the monastic and general populations [15] as markers for the prevention of cardiovascular dyshomeostasis, with the exception of profound hypovitaminosis-D in Orthodox monks, mainly due to their sartorial habits [11,12].

Additionally, the restriction of food intake in specific time-frames during the day has also been hypothesized to contribute to the benefits described above, a characteristic that hasattracted significant scientific and public interest during the last decade, through various intermittent-fasting patterns, practiced worldwide as a health-promoting diet [16]. Time-restricted eating (TRE) includes specific time-frames of food intake during the day, which vary from 4 to 12 h daily [e.g., 20 h of fasting vs. 4 h of permitted food intake—20:4—as well as additional time-frames (18:6, 16:8, etc.)] [17]. On the other hand, the impairment of the antioxidative capacity of the vascular endothelium is an established aggravating factor for the development of endothelial dysfunction and future major cardiovascular events [18,19]. On that basis, a considerable number of previous studies [20,21,22,23] have suggested that the MD is strongly associated with favorable effects on oxidative status, implying a potential pathway for exerting its well-established cardiovascular benefits. However, the results on COF as a vital subset of the MD and its effects on oxidative status, particularly compared to other healthy nutritional patterns widely adopted by the general population, remain scarce.

Additionally, these potential interactions have so far not been investigated in conjunction with other metabolic conditions associated with endothelial dysfunction, including impairment of vitamin D status and insulin resistance, particularly in vitamin D-deficient and overweight individuals. These results could elucidate the potential mechanisms of MD-related effects on antioxidative capacity and also elaborate on the research hypothesis, which indicates the macro- and micronutrient synthesis and increased intake of food antioxidants, rather than the timing of food intake, as the cornerstone of the attained metabolic benefits.

The present study attempted to evaluate the effects of COF in a group of vitamin D-deficient and overweight Orthodox nuns from Central and Northern Greece compared to the implementation of theTRE 16:8 dietary regimen in a cohort of adult women from the general population from the same region with regard to markers of oxidative status.

## 2. Methods

### 2.1. Design

This was a cross-sectional study after a period of 16-week implementation of COF and TRE in two groups of adult female nuns and lay women.

### 2.2. Study Population

We included 50 Christian Orthodox female adult nuns, from two different monasteries, 30–50 years of age, residing in Central and Northern Greece and an age-matched cohort of 50 adult lay women from the same region.

Orthodox nuns (but not lay women), with a baseline 25-hydroxyvitamin D concentrations ≥ 20 ng/mL (as initially evaluated from the same initial cohort—results published previously [12,13,14,15]) were excluded. Additional exclusion criteria for both groups were the following: body mass index (BMI) ≤ 25, amenorrhea ≥ 3 months, pregnancy, presence of chronic kidney disease, severe liver disease, diagnosis of prediabetes or diabetes mellitus according to ADA criteria, dyslipidemia, arterial hypertension, uncontrolled hypothyroidism, recent surgery, severe infections (during the past 3 months), administration of medications that can alter weight, glucose and lipid metabolism (e.g., statins, corticosteroids, and antipsychotics), intake of vitamins or mineral supplements, physical disabilities and/or neurodegenerative disorders that could affect physical activity, acute infections, and chronic degenerative diseases.

### 2.3. Dietary Patterns

Orthodox nuns with at least 16 weeks adherence to COF were included in the study, whereas women from the general population followed TRE for 16 weeks, after a wash-out period of 3 weeks, before inclusion in the study. Orthodox nuns followed the Athonian type of fasting as previously described [1,2,3,4], abstaining from consumption of animal products (meat, poultry, eggs, dairy, and cheese), with the exception of seafood and fish, which fasters were permitted to eat on two specific weekdays, while the general population group was allowed to eat low-fat meat products without specific distribution and cut-offs of macronutrients and daily caloric intake.

Orthodox nuns group adopted an 8 h eating interval (08:00 to 16:00), as dictated by typical monastery dietary rules, which are obligatory for all residents of the monastery, while TRE group consumed food from 09:00 to 17:00. Adherence to dietary plans was evaluated with a 3-day food record (two weekdays and one weekend day) at the end of the study period, while the Nutrition Analysis Software Food Processor [https://esha.com/products/food-processor/ (accessed on 2 August 2024)] [24] was used to analyze food records. Finally, levels, frequency, and duration of physical activity, divided into light, moderate, and intense physical activity, were recorded for all participants according to AHA recommendations [25].

### 2.4. Anthropometric Measurements and Biochemical Analysis

Anthropometric measurements and biochemical analyses were performed in both groups using standardized procedures. Exact methods, reference ranges, equipment used, and other details were previously analytically described [11]. In brief, body weight (BW) was recorded to the nearest 0.01 kg using a calibrated computerized digital balance (K-Tron P1-SR, Onrion LLC, Bergenfield, NJ, USA); each participant was barefoot and lightly dressed during measurement. BMI was calculated as the ratio of weight in kilograms divided by the height in meters squared (kg/m^2^) [26]. Body fat (BF) mass and percentage, visceral fat (VF), muscle mass, fat-free mass, and total body water were measured using bioelectrical impedance analysis (SC-330 S, Tanita Corporation, Tokyo, Japan) [27]. Blood samples were drawn in the morning, after a 12 h overnight fast by antecubital venipuncture, and the samples were stored at −20 °C prior to analysis. Samples were centrifuged and immediately frozen and then measured after one instance of defrosting, except from whole blood.

Calcium (Ca) concentrations were evaluated using the COBAS8000 automated analyzer system (Roche Diagnostics GmbH, Mannheim, Germany). Parathyroid hormone (PTH) and 25(OH)D were tested in the COBAS e 602 immunochemistry module using electro-chemiluminescence (ECL) technology (Roche Diagnostics GmbH, Mannheim, Germany). Reference ranges of values as well as inter- and intra-assay coefficients of variation for the examined parameters are as follows: Ca: 8.4–10.2 mg/dL, 0.8–1.3%, and 0.5–1.3%; PTH: 15–65 pg/mL (or 1.6–6.9 pmol/L), 1.1–2.0%, and 2.5–3.4%; 25(OH)D: ≥30 ng/mL, 2.2–6.8%, and 3.4–13.1%. Insulin resistance was calculated using the homeostasis model assessment (HOMA-IR) formula described by Matthews et al. [28] as follows: FPI (mU/mL) × FPG (mmol/L)/22.5, where FPI stands for fasting plasma insulin and FPG for fasting plasma glucose.

### 2.5. Markers of Oxidative Status

#### 2.5.1. Determination of Glutathione (GSH) Concentration in Red Blood Cells

GSH concentration was determined according to the method of Reddy et al. [29] as previously described [30]. At first, 400 μL of RBCL was mixed with 400 μL of 5% trichloroacetic acid (TCA), respectively, and centrifuged (1500× *g*, 5 min, 5 °C). Afterwards, 300 μL of the supernatant was mixed with 90 μL of 5% TCA and centrifuged (1500× *g*, 5 min, 5 °C). The samples were vortexed and incubated for 45 min in the dark at room temperature (RT), and the optical density was measured at 412 nm. GSH concentration was calculated based on the millimolar extinction coefficient of 2-nitro-5-thiobenzoate (TNB) (13.6 L/mmol/cm).

#### 2.5.2. Determination of Total Antioxidant Capacity (TAC) Concentrations in Plasma

TAC levels were evaluated based on the protocol of Janaszewska and Bartosz [31]. More elaborately, 20 μL of plasma was mixed with 480 μL or 460 μL of phosphate buffer (10 mM, pH = 7.4), respectively, and, immediately, 500 μL of 2,2-diphenyl-1-picrylhydrazyl radical (DPPH^•^) solution (0.1 mM) was added. The samples were vortexed, incubated for 1 h in the dark at RT, and centrifuged (1500× *g*, 3 min, 25 °C). Finally, the optical density was measured at 520 nm. TAC levels were expressed as the mmol of DPPH^•^ reduced to the corresponding hydrazine by the antioxidant compounds present in plasma or tissue homogenates.

#### 2.5.3. Determination of Thiobarbituric Acid Reactive Substances (TBARSs) Concentrations in Plasma

TBARS levels were determined by a slightly modified method by Keles et al. [32]. Specifically, 100 μL of plasma was mixed with 500 μL of Tris-HCl (200 mM, pH = 7.4) and 500 μL of 35% TCA and incubated for 10 min at RT. After that, 1 mL of sodium sulfate (Na_2_SO_4_) (2 M) and thiobarbituric acid (TBA) (55 mM) solution was added, and the samples were placed in a water bath for 45 min at 95 °C. The resulting supernatant was used to measure the optical density at 530 nm. TBARS levels were calculated by applying the molar extinction coefficient of malonyl dialdehyde (ΜDA) (156,000 L/mol/cm).

### 2.6. Ethical Considerations

The study was conducted in accordance with the Declaration of Helsinki on the human trial performance. Written informed consent for inclusion in the study was providedby participants. Official written approval for the inclusion of the Orthodox nuns group was providedby the Holy Supervision Council of the monasteries after submission of the full study protocol 12 months before study initiation.

### 2.7. Statistical Analysis

Continuous variables were reported as means and SDs. Dietary and nutrient intake were compared using paired samples *t*-test. Age differences between the groups with light, moderate, and intense physical activity were tested using one-way analysis of variance with Tukey post hoc test. The effect of level of physical activity on overall health markers was tested with analysis of covariance to control for age. Normality of distribution was tested with one sample Kolmogorov–Smirnov test (exact statistics).

The among-group comparison was undertaken using nonparametric Mann–Whitney U test. Linear regression was used for multi-adjusted analysis. Assumptions were checked for each statistical analysis. Level of significance was set at *p* < 0.05 (non-directional). Data were analyzed using SPSS v22.

## 3. Results

The Orthodox nuns were older than the lay women (median age 42 vs. 38, *p* < 0.001) but did not differ in median weight and BMI (Table 1). The groups did not differ in body fat (%), lean body mass (%), waist circumference, or degree of physical activity, with the exception of intense activity, in which the lay women reported higher rates. Regarding the nutritional analysis, the lay women consumed higher amounts of carbohydrates (g) (194.3 ± 23.4 vs. 159.6 ± 21.8) andtotal and saturated fat (24.4 ± 0.6 vs. 21.0 ± 0.1 and 16.4 ± 0.0 and 12.7 ± 0.0, respectively), whereas the Orthodox nuns reported higher amounts of protein and fiber intake (36.1± 0.8 vs. 24.2 ± 0.8).

**Figure 1:**
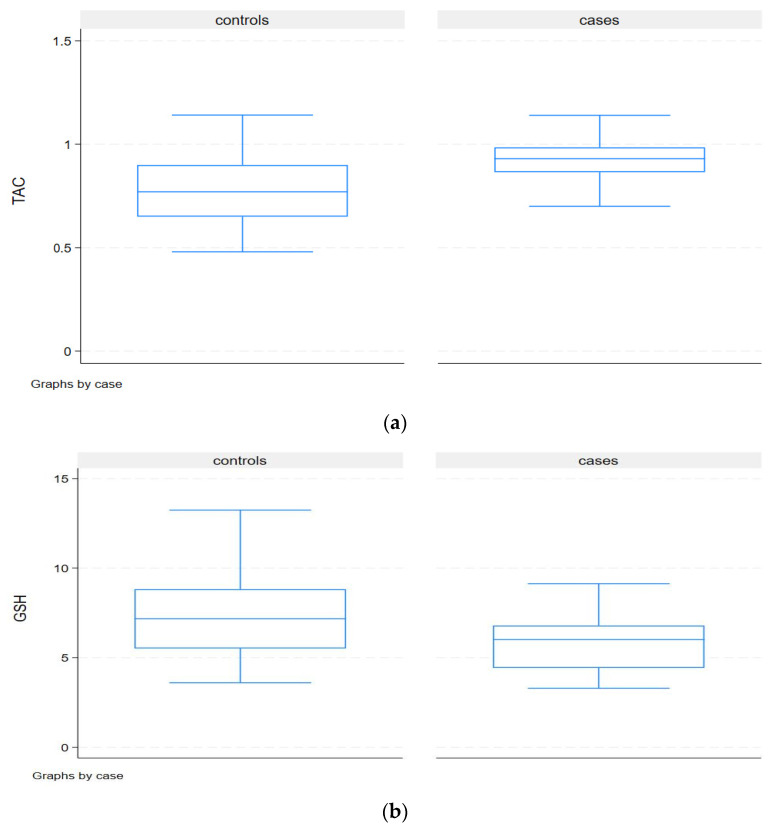
(**a**) Concentrations of TAC in Orthodox nuns (cases) and lay women group (controls), *p* < 0.001. Concentrations of TAC in Orthodox nuns (cases) and lay women group (controls). Orthodox nuns had a higher TAC concentration in serum (mean difference 0.19; 95% CI 0.13 to 0.26, *p* < 0.001), and, after adjusting for age, 25(OH)D concentrations, and BMI, nuns had a higher TAC concentration in serum (mean difference 0.21; 95% CI 0.15 to 0.27 *p* < 0.001). (**b**) Concentrations of GSH in Orthodox nuns (cases) and lay women (controls), *p* 0.04. Orthodox nuns had a lower GSH concentration in serum (mean difference −1.6; 95% significant (*p* = 0.45) with CI −2.6 to −0.7, *p* 0.001) compared to lay women, while the age 25(OH) concentrations and BMI effects were not significant.

Although expected, according to the study protocol, the hypovitaminosis D evident in the Orthodox nuns group resulted in significantly higher median serum PTH than among the lay women (45.6 vs. 19.4, *p* < 0.001) after adjusting for seasonal variation (Table 2). In addition, after adjusting for age and the 25(OH)D3 concentrations in the linear regression across all the patients, PTH had a significant positive association with age (+6.0 pg/mL per 10-year increase in age, *p* < 0.001) and a significant negative association with serum 25(OH) D3 status (–0.61 pg/mL per ng/mL increase in serum D3). The Orthodox nuns demonstrated lower median fasting insulin concentrations (5.3 vs. 7.2, *p* 0.02) compared to the lay women, and, even after adjusting for age and BMI, the difference remained significant. Of major interest is the fact that the insulin concentrations lacked a significant association with BMI or age in both groups. Regarding the redox status, the Orthodox nuns manifested a lower median GSH compared to the controls (6.0 vs. 7.2, *p* < 0.04) and a higher median TAC (0.92 vs. 0.77, *p* < 0.001). The TBARS comparisons showed no significant difference between the two groups. After adjusting for age in the linear regression, the Orthodox nuns had a lower GSH concentration in the serum (mean difference −1.7; 95% CI −2.7 to −0.7, *p* < 0.001) compared to the controls, while the age effect was not significant (*p* = 0.45). After adjusting for age, BMI, and the25(OH)D concentrations in the linear regression, the nuns had a higher TAC concentration in the serum (mean difference 0.19; 95% CI 0.13 to 0.26, *p* < 0.001), whereas, after adjusting for age, BMI, and the 25(OH)D concentrations in the linear regression, the nuns had a lower GSH concentration in the serum (mean difference −1.6; 95% significant (*p* = 0.45) with CI −2.6 to −0.7, *p* < 0.001) compared to the lay women, while the age and BMI effects were not significant (Figure 1). After adjusting for age and BMI, the nuns had a higher TAC concentration in the serum (mean difference 0.21; 95% CI 0.15 to 0.27 *p* < 0.001), age, BMI, and total fat; the effects were not significant. No significant associations of oxidative status with the 25(OH)D concentrations, PTH, and markers of glucose homeostasis were evident.

## 4. Discussion

The MD is a plant-based diet, rich in fruit, vegetables, nuts, and herbs, with fewer fish and dairy products and with less red meat and red wine. The MD includes various nutritional compounds, with well-established beneficial effects on oxidative status. A plethora of previous basic and clinical studies have suggested that the MD has been shown to be one of the healthiest eating patterns, with various metabolic benefits, partly mediated through its antioxidant capacity [33,34]. Dai et al. studied the ratio of reduced to oxidized glutathione (GSH/GSSG) in twins. The higher the ratio, the lower the oxidative stress, providingthe result of a higher ratio up to 7% in those individuals who followed the Mediterranean diet regardless of the adjustment of the energy intake [35]. In a sub-cohort of the PREDIMED trial, those participants with high cardiovascular risk were randomized to a Mediterranean diet supplemented with extra-virgin olive oil and manifested significant reductions in their cellular lipid levels and lipid oxidation, as well as the malondialdehyde concentrations in mononuclear cells, without changes in their serum glutathione peroxidase activity [36].

The documented benefits of the MD include the consumption of unsaturated fatty acids, found in olive oil, which contain 3,3-dimethyl-1-butanol, thus preventing the formation of trimethylamine-1-oxide, one of the oxidants related to cardiovascular events [37,38].

Additionally, the MD synthesis is rich in oleicacid and alpha-linoleicacid, found in nuts, fruit and vegetable flavonoids, as well as omega-3-polyunsaturatedfattyacids, and fiber and polyphenols, all of which have antioxidative, anti-bacterial, and anti-inflammatory effects [39,40,41]. Moreover, wholegrains, as a vital compound of the MD, contain a polyaminecalled spermidine, which has been shown to extend the chronological life-span in flies, nematodes, rodents, and human cells. Spermidine is known to inhibit histone acetyltransferases, which results in higher resistance to oxidative stress [42].

TRE has also been the objective of recent studies regarding its potential beneficial effects on cardiometabolic health. Given the fact that hormones undergo a circadian rhythm, metabolic and stress hormones such as insulin, cortisol, growth hormone, and melatonin undergo the same variations, providing different levels between a calorie-restricting diet and intermittent-fasting diet, which restricts the feeding time tocertain hours [43]. Mc Allister et al. studied the impact of intermittent fasting on the markers of cardiometabolic health, measuring several markers of inflammation, oxidative stress, and cardiometabolic health (insulin, ghrelin, leptin, glucagon, adiponectin, resistin, advanced glycatedend products (AGE), advanced oxidation protein products, total nitrite–nitrate levels, tumor necrosis factor-α, and interleukins (IL)-6, IL-8, and IL-10) and showed that time-restricted feeding resulted in significant reductions in the advanced oxidation protein products (~31%) and AGEs (~25%); however, no other changes were found [44]. Recent randomized clinical trials also demonstrated that a6h feeding period for 5 weeks improved insulin sensitivity, β-cell responsiveness, and oxidative stress irrespective of weight loss [45]. These results were also previously confirmed by other groups, where4 h and 6h TRE for 5 weeks resulted in a reduction in8-isoprostane as a marker of oxidative stress to lipids,4-hydroxynonenal adducts, protein carbonyls, and nitrotyrosine [46].

COF is a plant-based subset of the traditional MD followed for more than a thousand years bya large part of the Greek Orthodox general population for religious purposes from 90 to 150 days per year. Greek Orthodox monasteries adhere to this dietary regimen throughout the year, with the addition of TRE (usually 16:8) characteristics in their daily dietary regimen, which is strictly followed by all the members of the monasterial community, comprising an optimal sample for nutritional studies.

To our knowledge, this is the first cross-sectional study reporting preliminary results on the comparative effects of COF on the oxidative markers in vitamin D-deficient Greek Orthodox nuns and the TRE (16:8) dietary regimen in a group of lay women with vitamin D sufficiency. These results indicated (i) increased antioxidative capacity (TAC) in the group of Orthodox nuns after a 16-week period of COF compared to a 16:8 TRE and (ii) increased GSH levels in the lay women group compared to the group of Orthodox nuns as well as comparable TBARS levels in both groups after adjusting for several confounders, which suggest potential diverse effects of COF and TRE on oxidative status.

We have repeatedly reported on the effects on COF on body weight, lipid parameters, adipokines, and vitamin D status regarding the existence of severe hypovitaminosis D in Orthodox male monks, mainly due to their sartorial habits.

However, this is the first report on the effects of COF on oxidative equilibrium, particularly compared to a health-promoting pattern like TRE.

Our research hypothesis raised the question regarding thenon-inferiority of TRE compared to COF in a vitamin D-deficient monastic population (as in most similar monastic communities in Greece) taking into account that the women included in the TREregimen were not instructed to follow an MD-specific dietary pattern. According to previous results, chronic vitamin D deficiency is a state of increased oxidative stress, which reduces the capacity of mitochondrial respiration through modulating the nuclear mRNA down regulating the expression of complex I of the electron transport chain, thus reducing adenosine triphosphate (ATP), resulting in increased formation of ROS, and augmenting oxidative stress [47].

Maintaining optimum levels of redox biomarkers is crucial for preventing oxidative damage, supporting detoxification processes, and ensuring proper immune function. The previous literature proposed that the clustering of high and low GSH levels might provide strong causality for type 2 diabetes and metabolic syndrome [47]. Our results failed to suggest the superiority of COF over TRE in the group with the confirmed MD-type dietary regimen and TRE characteristics of Orthodox nuns compared to the 16:8 regimen without specific MD-related dietary characteristics.

A plausible explanation could be that the general population following a TRE pattern complies with a healthy dietary pattern, which, despite not being identical to the MD, also exerts benefits on the GSH concentrations, always taking into account the limitations of this study. Another explanation could be related to the potential adverse effects of hypovitaminosis D, evident in the Orthodox nuns included in this study, on the GSH concentrations, as previously reported [12]. Vitamin D supplementation in this group of vitamin D-deficient nuns could elucidate this potential biological association regardingGSH status. TRE could also have independent beneficial effects on oxidative status, which are evident without strict adherence to an MD-related pattern, as previously reported [12]. Finally, our study failed to establish an association betweenimpaired vitamin D status and oxidative markers, which could be attributed to its cross-sectional design. This study has several limitations and can only be considered as a pilot study, with findings thatdefinitely require confirmation in a prospective study. In detail, the number of included participants was relatively small; however, this is a representative sample of Orthodox nuns according to their dietary and physical activity plans. We have also not included a detailed analysis regarding the intake of the dietary antioxidants in the two groups, which could explain the diversity in the markers of oxidative status. In addition, the inclusion of a non-nutritionally restricted control group without vitamin D deficiency might have strengthened the analysis.

Finally, since no baseline evaluation, prior to the implementation of the dietary interventions, was feasible for both groups, we were unable to establish causal associations.

## 5. Conclusions

In conclusion, the results of this small pilot study indicate that both dietary regimens have advantages regarding oxidative markers compared to each other, with increased TAC in the group of Orthodox nuns after a 16-week period of COF in comparison to the increased GSH concentrations in the lay women group following the 16:8 TRE, and comparable concentrations of TBARSs. Future randomized trials are required to investigate the superiority or non-inferiority between these dietary patterns in the daily clinical setting.

## Figures and Tables

**Table 1:** Demographics of Orthodox nuns compared to lay women.

	Orthodox Nuns (*n* = 50)	Lay Women (*n* = 50)	*p*
Demographics			
Age (years)	42 (36–50)	38 (34–42)	0.03
Weight (kg)	71.5 (64–82)	66 (60–87)	0.31
BMI (kg/m^2^)	27.0 (24.2–29.0)	26.8 (22.0–32.0)	0.19
Body fat (%)	24.5 ± 9.4	22.1 ± 8.1	0.23
Lean body mass (%)	39.9 ± 6.3	41.2 ± 7.1	0.15
Waist circumference (cm)	92.4	89.1	0.11
Physical activity			
Light	N = 9	N = 7	0.31
Moderate	N = 27	N = 25	0.48
Intense	N = 14	N = 18	0.03
Years of monasticism	10.5 ± 9.8	-	-
Deaconship of Orthodox Nuns	Baker (3); Botanist (2); Cook (5); Cooking assistant (5); Dining assistant (5); Ecclesiastical chanter (6); Gardener (3); Housekeeper (3); Iconographer (6); Laundry assistant (4); Pharmacist (2)		

Orthodox nuns were older than lay women (median age 42 vs. 38, *p* < 0.001) but did not differ in median weight and BMI. Comparison of nuns and lay women did not show differences in body fat (%), lean body mass (%) and waist circumference, as well as degrees of physical activity, with the exception of intense activity, in which lay women reported higher rates. Age differences between the groups with light, moderate and intense physical activity were tested using one-way analysis of variance with Tukey post hoc test. The effect of level of physical activity on overall health markers was tested with analysis of covariance to control for age. Normality of distribution was tested with one sample Kolmogorov–Smirnov test.

**Table 2:** Nutritional habits and oxidative stress markers comparison between Orthodox nuns and lay women.

Energy (kcal)	1565.9 ± 64.5	1890.0 ± 71.0	<0.01
Carbohydrates (g)	159.6 ± 21.8	194.3 ± 23.4	0.03
Protein (g)	89.2 ± 1.3	72.3 ± 1.3	0.04
Daily fat intake (g)	21.0 ± 0.1	24.4 ± 0.6	0.02
Daily saturated fat intake (g)	12.7 ± 0.0	16.4 ± 0.0	0.01
Total fiber intake (g)	36.1 ± 0.8	24.2 ± 0.8	0.02
25-hydroxy-vitamin D3 (ng/mL)	15.7 (11.4–19.8)	26.1 (18.2–31.9)	0.02
PTH (pg/mL)	45.6 (39.6–54.7)	19.4 (13.1–28.5)	<0.001
Calcium (mg/dL)	9.4 (9.1–9.7)	9.1 (8.8–9.3)	0.15
Insulin (IU/L)	5.3 (3.4–6.7)	7.1 (4.7–11)	0.02
Fasting glucose (mg/dL)	84.4 ± 10.1	89.2 ± 9.7	0.43
HOMA-IR	1.02 ± 0.4	1.26 ± 0.7	0.21
Oxidative status			
TAC	0.93 (0.87–0.99)	0.77 (0.65–0.90)	<0.001
GSH	6.0 (4.4–6.8)	7.2 (5.5–8.8)	0.04
TBARSs	7.3 (5.8–8.3)	7.6 (6.9–8.4)	0.28

Comparison of the nutritional habits of Orthodox nuns to lay women showed that lay women consumed higher amounts of carbohydrates (gr) (194.3 ± 23.4 vs. 159.6 ± 21.8) and total and saturated fat (24.4 ± 0.6 vs. 21.0 ± 0.1 and 16.4 ± 0.0 and 12.7 ± 0.0, respectively), whereas Orthodox nuns reported higher amounts of protein and fiber intake (36.1 ± 0.8 vs. 24.2 ± 0.8). Dietary and nutrient intake were compared using paired samples *t*-test.

## Data Availability

The original contributions presented in the study are included in the article, further inquiries can be directed to the corresponding author.
